# Magnetic circuit design for the performance experiment of shear yield stress enhanced by compression of magnetorheological fluids

**DOI:** 10.1038/s41598-024-51413-z

**Published:** 2024-01-07

**Authors:** Cheng Bi, Erda Bi, Hongyun Wang, Chunlin Deng, Huixin Chen, Yun Wang

**Affiliations:** 1 School of Intelligent Manufacture, Taizhou University, Taizhou, 318000 Zhejiang China; 2 HD Ningbo School, Ningbo, 315010 Zhejiang China

**Keywords:** Engineering, Materials science, Nanoscience and technology

## Abstract

The shear yield stress is an important parameter for the industrial application of magnetorheological (MR) fluids. A test equipment was designed and built to perform investigations on the behaviours of compression and shear after squeeze of MR fluids. Mathematical expression of magnetic flux density was further established. Furthermore, the magnetic field distribution of the test device based on two-coil mode and single-coil mode was simulated and compared using finite element analysis(ANSYS/Multiphysics). An experimental test system was fabricated and modified based on the final conditions and simulation results. The compression and shear after squeeze performances of MR fluids were tested. The results showed that a smaller initial gap distance or a larger compressive strain corresponds to a larger compressive stress under the same external magnetic field strength. The shear yield stress after the squeeze of MR fluids increases quickly with the increasing compression stress and the increasing magnetic flux density. This test equipment was thought to be suitable for studying the compression and shear after squeeze performances of MR fluids.

## Introduction

Magnetorheological (MR) fluids are smart materials whose rheological properties can be reversibly changed at a millisecond-level by the applied magnetic field^1,2^. MR fluids can be changed from a Newtonian fluid into a Bingham fluid by the applied magnetic field, which shows field-induced yield stress of MR fluids^3^. Potential applications of MR fluids in the industry, such as dampers, clutches/brakes, and actuators, have been suggested^4–6^. However, the real applications listed above of MR fluids based on shear mode are restricted because of the lower shear yield stress (50–120 kPa)^4^. For example, the reported torque of MR brake with shear mode geometry is usually lower than 150 Nm, while the torque of general automotive brakes is at least 1000 Nm, which is not high enough for many real applications.

So, a lot of work has been performed to look for other ways to enhance the yield stress of MR fluids in recent decades. On the one hand, various new types of MR fluids with high shear stress have been developed^7^. On the other hand, compressive mechanical properties in squeeze mode have been found to have higher yield stress than that in shear mode^8–11^. Tang et al. first found that the formation of thick strong columns of MR fluids under compression results in the enhancement of the yield shear stress, which is called the squeeze-strengthening effect^8^. The compressive properties of MR fluids in squeeze mode have been studied extensively. The influence factors of the compressive force/stress for MR fluids including magnetic field strength, initial gap distance, compressive strain, viscosity of carrier fluid, and particle volume concentration, have been widely discussed^1,11–14^. Theoretical models have been proposed for the prediction of the compressive properties of MR fluids in squeeze mode^15–18^.

The above-reported compressive behavior literature of MR fluids was studied based on the case of the constant area^1,12,13,15,18^ or the constant volume^16^ operation by self-assembled devices^16,18^ or commercial setups^1,12,13,15,17,19^. Even though MR fluids have been investigated repeatedly in squeeze mode, there are few studies on the shear yield stress of MR fluids after squeeze. Tang et al. compressed the MR fluid before a shear force is applied, and found that the shear yield stress of MR fluids after squeeze is ten times that under shear mode under moderate magnetic field by a self-assembled device^8^. At the applied magnetic field, chain structures including many incomplete chains form in milliseconds and the weakness of chain structure is at the ends of the chain. Due to the wall effect, the ends of the chain are more easily broken under shear^9^. However, the ends of the chain can be strengthened because of the formation of thick columns during compression^8,9^. Meanwhile, incomplete chains can be repaired during compression^9^. Moreover, when the distance between particles is very close after the squeeze, the great enhancement of yield stress is mainly due to the aggregation effect of the chain structure, especially the friction effect^9,10^. However, See et al. have discovered that the shear yield stress of MR fluids after squeeze could not be improved^20^. Afterwards, Mazlan et al. found the compressive stress when the initial gap distance is small is lower than that when the initial gap distance is large^11,18^. These researches are contrary to the results for ER fluids^21^ and MR fluids^8,19,22^. Therefore, there is still an argument about whether the compressive resistance of MR fluids after squeezing can be improved. The stable column/BCC structures of MR fluids after squeezing by SEM and optical microscopy have been observed^19^, but it is still difficult for establishing the precise mathematical model including various influencing factors, such as applied shear strain, magnetic field, compressive force, initial gap distance, compressive strain, volume fraction, to predict the shear after the squeeze behavior of MR fluids. Under the quasi-static compression, the MR fluid is often described as linear, uniform and anisotropic permeability continuous medium. Nevertheless, the specific form of the constitutive equation can not be deduced only based on the continuum media theory because the constitutive equation of MR fluids is physical in nature and it should be determined by the structural properties of MR fluids. The performance of compression and shear after squeeze of MR fluids can be studied with existing commercial rheometers. However, it is still a great challenge for commercial rheometers to study the properties of performance of compression and shear after squeeze of MR fluids because the maximum compressive force of the general commercial rheometer, such as Anton Paar MCR 301, is 50 N. The compressive force has reached the limit value when the compressive strain is smaller under the high magnetic flux intensity. The high shear yield stress enhanced by compression of the MR fluid shows a potential for developing high-performance MR damper, MR clutch, and MR mounts. Therefore, the relationship between compressive force and the shear yield stress needs further to be reported or discussed under higher magnetic field or larger compressive strain.

In this study, a self-assembled test device with constant area was constructed to investigate the performance of compression and shear after squeeze. Mathematical expressions of magnetic flux density were further established. Magnetic field excitation devices based on two-coil mode and single-coil mode were compared. Furthermore, the magnetic field distribution in the test device was simulated using finite element analysis(ANSYS/Multiphysics). The performance of compression and shear after squeeze were studied under variable factors. The test results are discussed. The enhanced shear yield stress after squeezing is attributed to the formation of more stable structures and the friction between particles due to the direct mechanical contact during compression.

## Design and simulation of the experimental device

### Design of the experimental device

**Figure 1:**
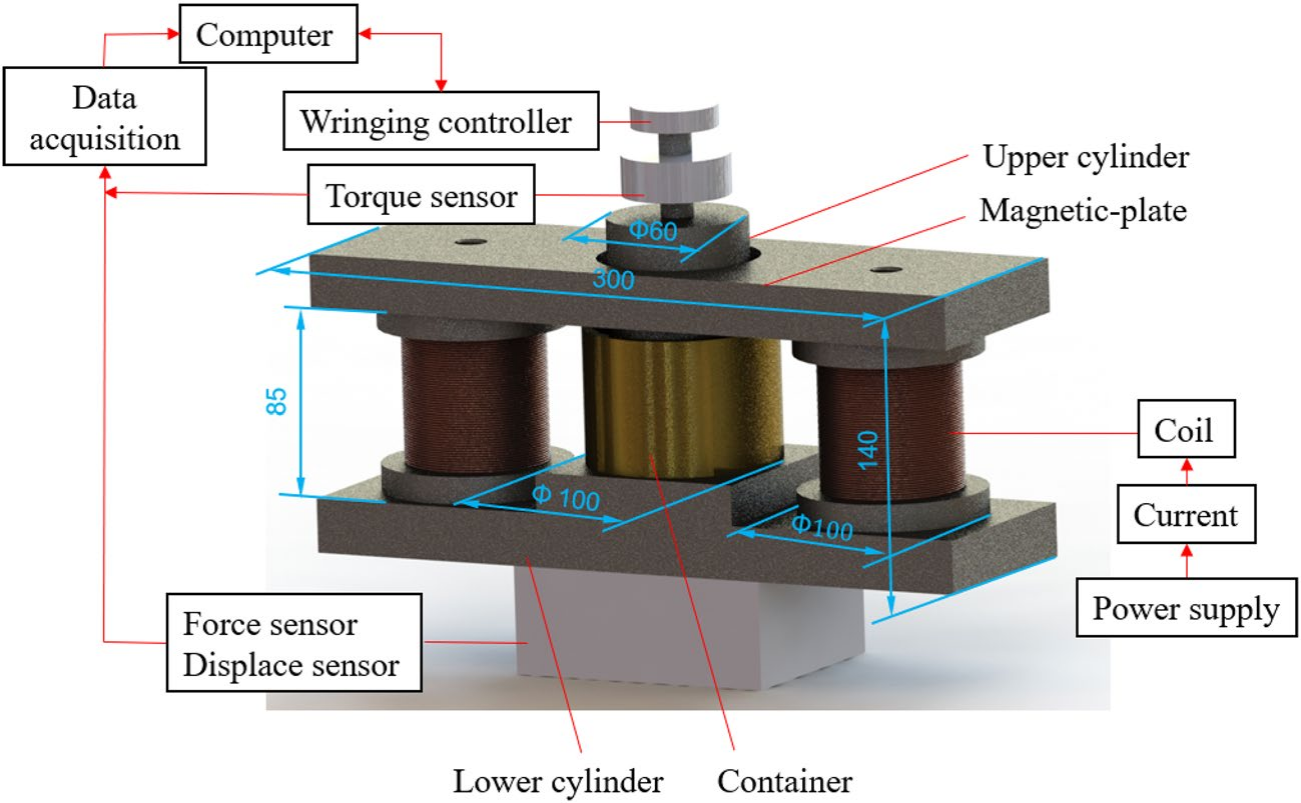
The schematic diagram of the experimental setup.

A schematic diagram of the experimental device is shown in Fig. 1. The container made of copper between the upper and lower plates is filled with the MR fluid. The diameter of the upper plate and the inner diameter of the container is 60 mm and 61 mm, respectively. The gap region between the upper plate and the container is to allow the MR fluid to overflow during compression. The coils wrapped around the iron core are placed on the side of the copper container. The force sensor, the displacement sensor, and torque sensor have a measurement range of 0–1.5 t, 0–12.5 mm, and 100 N·m, respectively. The three sensors are connected to the USB-1608FS data acquisition card in parallel and then sampled by the computer.

### Design and simulation of the magnetic circuit

Generating the high strength and uniform magnetic flux density in the MR fluid is the primary purpose for the design of magnetic circuit^18^. Therefore, the role of the electromagnetic excitation device is to concentrate the magnetic flux density produced by the coil and direct it from the iron core to pass through the MR fluid. In order to form a closed magnetic loop, the container made of copper was chosen to stop the magnetic flux from across the side of the container. The electromagnetic pure iron of brand DT4C due to its high magnetic permeability (μ) and saturation magnetic induction (Bs) was chosen to make the iron core, the concentrating flux plate, the upper plate, and the lower plate.

**Figure 2:**
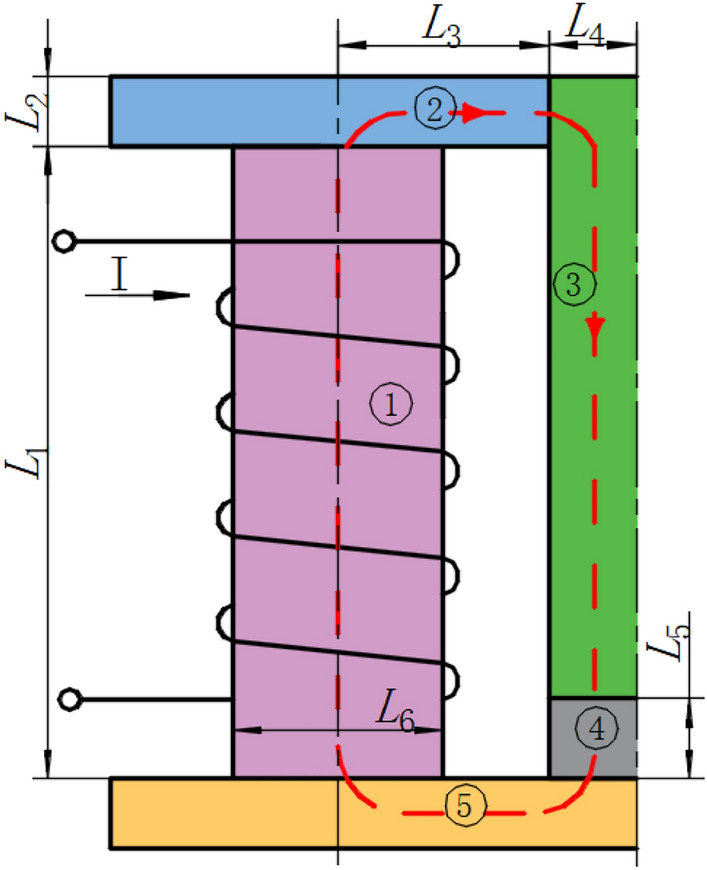
Magnetic circuit of the proposed electromagnetic excitation device.

According to Ohm's law, the magnetic reluctance can be described as

1 $$R_{i} = \frac{{L_{i} }}{{\mu_{i} S_{i} }}$$

where *μ*_*i*_ is the magnetic permeability in the *i* region; *L*_*i*_ is the length of the magnetic center line in the *i* region; *S*_*i*_ is the average sectional area of magnetic flux in the *i* region. The total magnetic reluctance of the proposed electromagnetic excitation device is divided into five regions, as shown in Fig. 2. According to Eq. (1), *R*_*1*_, *R*_*2*_, *R*_*3*_, *R*_*4*_, and *R*_*5*_ can be derived respectively as:

2 $$R_{1} = \frac{{4L_{1} }}{{\pi \mu_{0} \mu_{r} L_{6}^{2} }},\;R_{2} = \frac{{L_{3} }}{{\mu_{0} \mu_{r} WL_{3} }},\;R_{3} = \frac{{L_{1} + L_{2} - L_{5} }}{{\pi \mu_{0} \mu_{r} L_{4}^{2} }},\;R_{4} = \frac{{2L_{5} }}{{\pi \mu_{0} \mu_{m} L_{4}^{2} }},R_{5} = \frac{{L_{3} + L_{4} }}{{\mu_{0} \mu_{r} W\left( {L_{3} + L_{4} } \right)}}\;$$

where *μ*_*0*_ is the air permeability; *μ*_*r*_ is the magnetic permeability of the electromagnetic pure iron; *μ*_*m*_ is the magnetic permeability of MR fluid; *W* is the width of the magnetic pole. According to the connection in the series theorem of the magnetic circuit, the total magnetic reluctance *R*_*T*_ is

3 $$R_{T} = R_{1} + \;R_{2} + \;R_{3} + \;R_{4} + \;R_{5}$$

Applying Kirchhoff’s second law, the magnitude of the magnetic flux *Φ* can be represented as:

4 $$\sum {\Phi R}_{m} = \sum {NI} = \sum {HL}$$

where *N* is the number of turns in the coil, *I* is the applied current in a coil, and *H* is the magnetic field strength*.* For a magnetic circuit with branches, the same magnetic field direction, Eq. (4) can be written as

5 $$NI = \Phi_{1} R_{1} + \Phi_{2} R_{2} + \Phi_{3} R_{3} + \Phi_{4} R_{4} + \Phi_{5} R_{5}$$

Considering the magnetic circuit with an air gap, the magnetic leakage coefficient *ξ*_*i*_ can be introduced. The magnetic fluxes in different regions can be derived as

6 $$\Phi_{1} = \xi_{2} \Phi_{2} = \xi_{3} \Phi_{3} = \xi_{4} \Phi_{4} = \xi_{5} \Phi_{5}$$

where the value *ξ*_*i*_ is different according to the shape/size of the magnetic circuit and it is often in the range of 2–2.5^23,24^. Therefore, according to Eqs. (5) and (6), the magnetic flux in the MR fluid region *Φ*_*5*_ can be calculated as

7 $$\Phi_{5} = \frac{IN}{{R_{t} }}$$

where *R*_*t*_ is defined by

8 $$R_{t} = \xi_{5} R_{1} + \frac{{\xi_{5} }}{{\xi_{2} }}R_{2} + \frac{{\xi_{5} }}{{\xi_{3} }}R_{3} + \frac{{\xi_{5} }}{{\xi_{4} }}R_{4} + R_{5}$$

The magnitude of the magnetic flux *Φ* can also be represented as:

8 $$\Phi =BS$$

where *B* is the magnetic flux density. Therefore, the magnetic flux density in the MR fluid region *B*_*5*_ can be calculated by

10 $$B_{5} = \frac{{\Phi_{5} }}{{S_{5} }} = \frac{2NI}{{\pi L_{4}^{2} R_{t} }}$$

**Figure 3:**
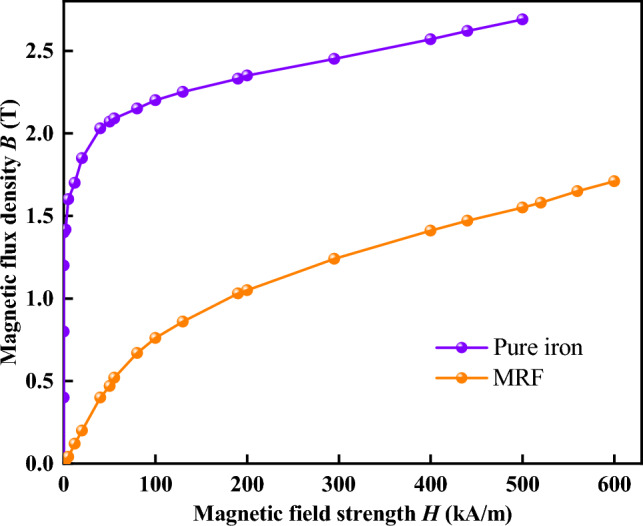
Magnetic induction curve for MRF-2035 and pure iron.

Equation (10) shows that *B*_5_ is proportional to the product of *N* and *I* in the magnetic circuit. Therefore, the enameled wire specification, number of turns*,* the value of applied current, and the size of each structure of the magnetic circuit can be determined according to Eq. (10). In the design, the maximum magnetic flux density in the MR fluid is 0.6 T. Then, the core diameter and its height are determined as 65 mm and 65 mm, respectively. The number of coil turns on each core and the diameter of the coil are determined as 1300 and 0.991 mm, respectively. The current density is limited to 5.85 A/mm^2^ and the maximum applied current is 2.5 A. The magnetic permeability of copper and air materials is μ = 0.99979 and μ = 1, respectively, so they are considered non-magnetic materials. Electromagnetic pure iron and MR fluid were assumed to be magnetic materials and their magnetic properties were considered to follow the B–H curves, as shown in Fig. 3. The main parameters of the electromagnetic excitation device are presented in Table 1.

**Table 1:** Main parameters of the electromagnetic excitation device.

Parameters	Values
Diameter of the upper plate	60 mm
Inner diameter of the container	61 mm
Diameter of core	65 mm
Height of core	65 mm
Turn of coil	1300 N
Diameter of coil	0.991 mm
Maximum of applied current	2.5 A
Maximum of magnetic flux density in MR fluids	0.6 T

**Figure 4:**
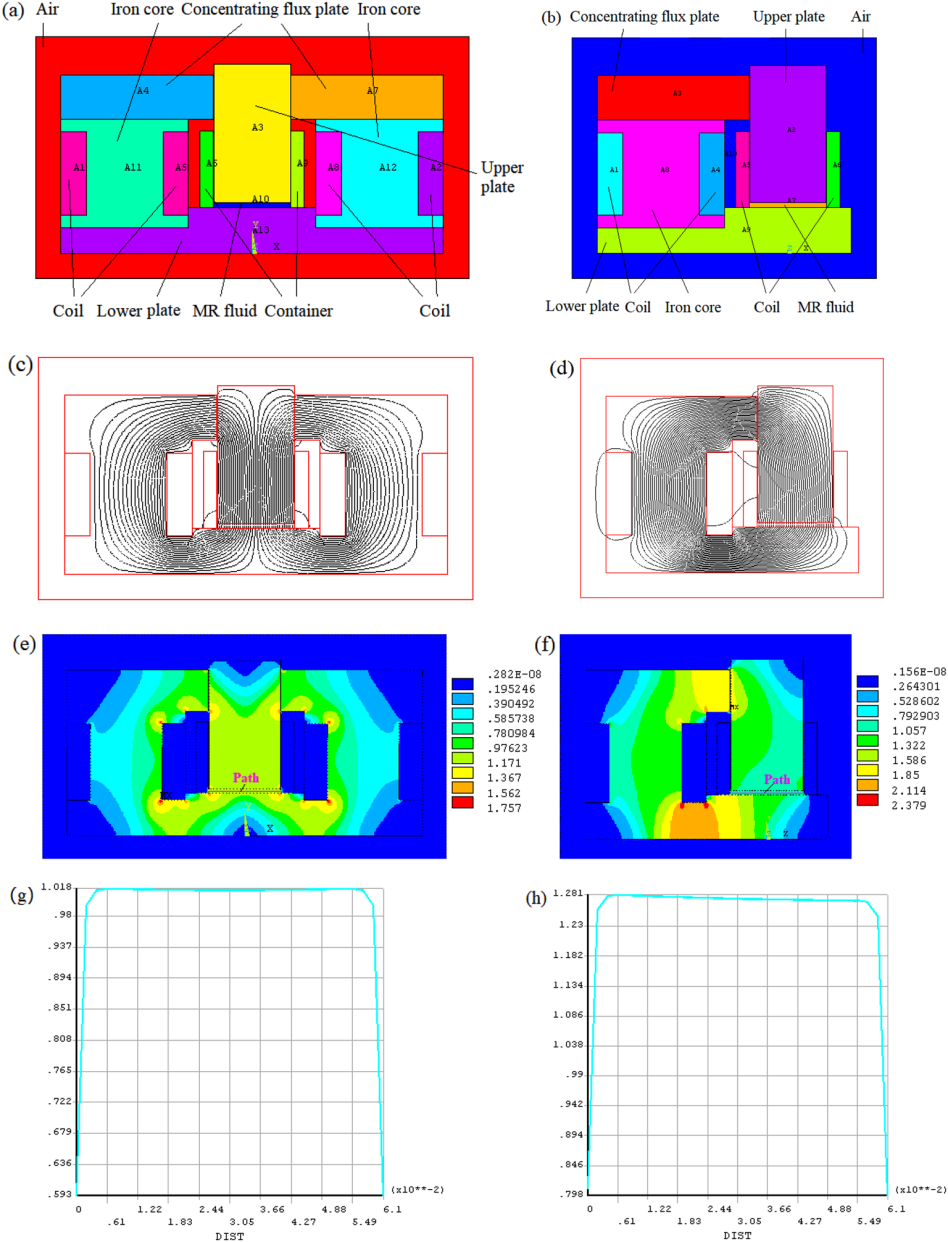
Simplified finite element model of the test device for (**a**) two-coil mode (**b**) single-coil mode (**c**) magnetic line distribution based on two-coil mode (**d**) magnetic line distribution based on single-coil mode (**e**) magnetic flux density distributions based on two-coil mode (**f**) magnetic flux density distributions based on single-coil mode (**g**) magnetic flux density versus the paths based on two-coil mode (**h**) magnetic flux density versus the paths based on single-coil mode.

To produce the high and uniform magnetic flux density in MR fluids, two designs of electromagnetic excitation devices were proposed, namely two-coil mode and single-coil mode. ANSYS/Multiphysics was used to analyze the 2D magnetic behavior of the test device, as shown in Fig. 4. It is necessary to simplify the model of the test device when the ANSYS software is used to analyze the magnetic field of each model. Figure 4a,b show the simplified two-coil mode and single-coil mode, respectively, where all threaded holes on the test device are ignored and the magnetic guide plate and the base are considered as one unit. The magnetic line distribution based on two-coil mode and single-coil mode in the test device is shown in Fig. 4c,d, respectively, when the applied current was 2.5 A, and the initial gap distance was set to 0.5 mm. It can be seen most of the magnetic lines pass vertically through the gap of MR fluid. And the magnetic flux distribution in the two-coil mode is more uniform than that in the single-coil mode. The magnetic flux density distributions based on two-coil mode and single-coil mode in the test device are shown in Fig. 4e,f, respectively. To observe the magnetic field distribution through the MR fluid, an observation path is made from the left to the right end of the MR fluid along the inner diameter of the container, as shown in Fig. 4e. The curves of the magnetic flux density versus the paths based on the two-coil mode and the single-coil mode in the MR fluid are shown in Fig. 4g,h, respectively. They show how the flux density varies with the path. Moreover, the distribution of magnetic flux density based on two-coil mode is more uniform in the middle of the path than that based on the single-coil mode. Figure 4 shows that the design based on the two-coil mode is better than that based on the single-coil mode. Therefore, the two-coil mode is adopted in the design of an electromagnetic excitation device.

**Figure 5:**
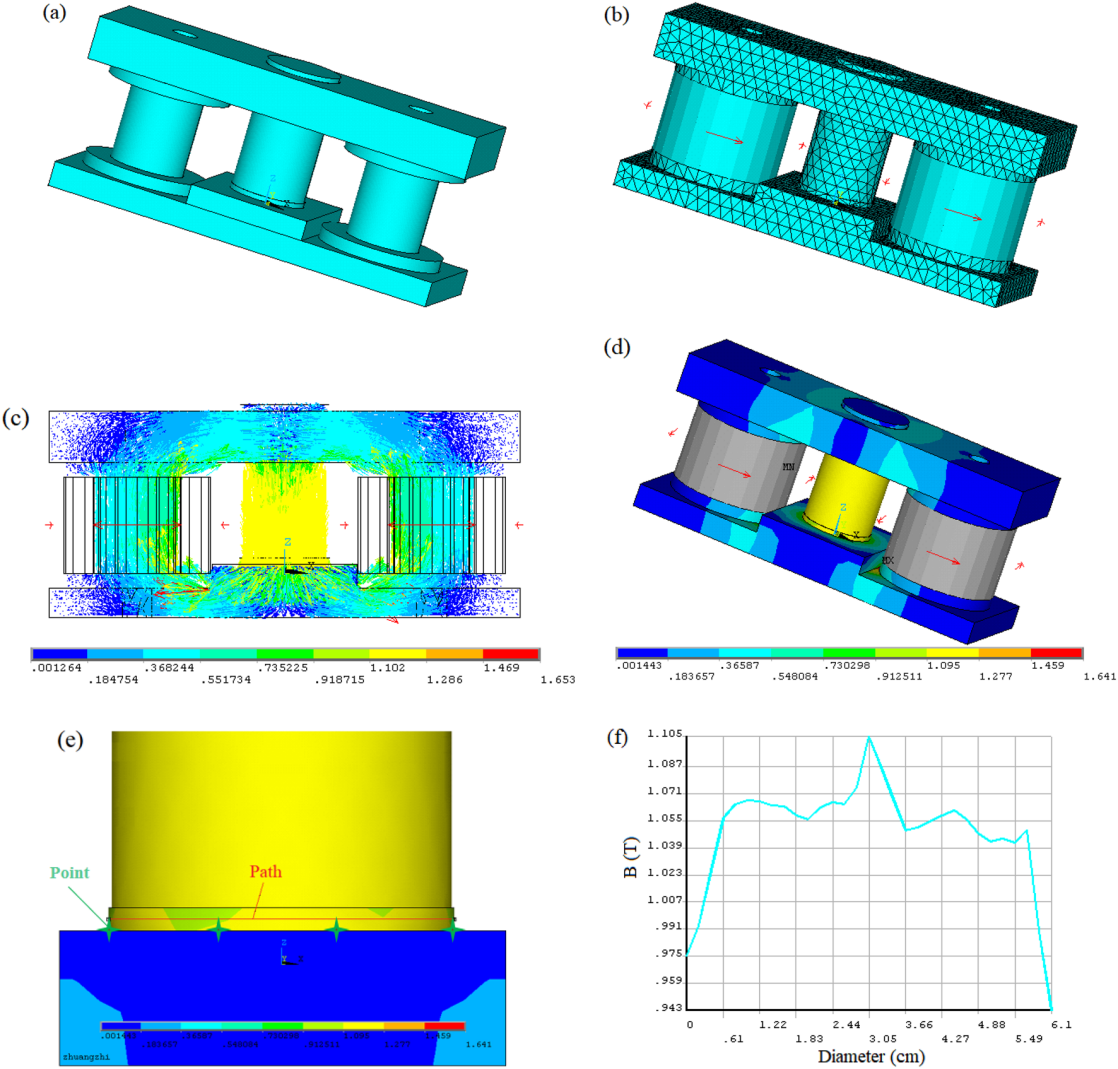
3D magnetic field analysis in the test device based on two-coil mode (**a**) simplified coil model (**b**) mesh model (**c**) vector graph of the magnetic induction intensity (**d**) magnetic flux density distribution (**e**) partial enlarged detail of observational path for the MR fluid gap (**f**) magnetic flux density changed with the path in the MR fluid at the applied current of 2.5 A and the initial gap distance of 0.5 mm.

To more clearly observe the magnetic field distribution through the MR fluid, it is necessary to conduct 3D magnetic field analysis for the test device based on two-coil mode. The 3D magnetic field analysis in the test device by ANSYS is shown in Fig. 5. Figure 5a,b show the simplified coil model and mesh model, respectively. In a 3D electromagnetic field analysis, the distribution of magnetic lines is represented by the vector of magnetic induction intensity. Figure 5c shows the vector graph of the magnetic induction intensity. Figure 5d shows the magnetic flux density distribution through the whole device. Similarly, an observational path was made from the left to the right end in the MR fluid, as shown in Fig. 5e. Figure 5f shows the curves of the magnetic flux density versus the paths in the MR fluid gap. It indicates that the magnetic flux density first increases precipitously in the path range of 0–6.1 mm, and then basically keeps unchanged in the path range of 6.1–26.9 mm, and at last, rapidly increases to a maximum of 1.105 T at the path of 30.5 mm. At the paths range of 30.5–61 mm, the curve of the magnetic flux density shows an opposite trend. This also shows that the magnetic flux density is perfect in the path range of 6.1–56.5 mm.

**Figure 6:**
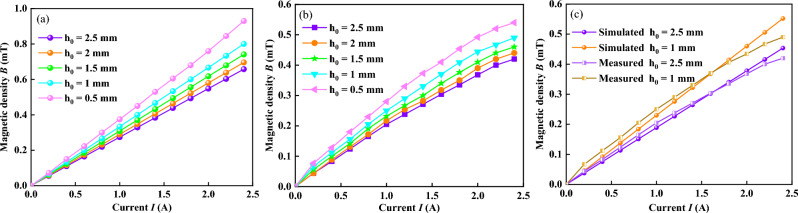
(**a**) Simulated (**b**) measured (**c**) comparison of simulated and measured magnetic flux density versus current at different initial gap distances.

The magnetic flux density generated by the coil could be measured using a tesla meter. It is the average value of that simulated or measured under the six-point method obtained along the edge of the upper plate, as shown in Fig. 5e. Figure 6 shows the simulated and the measured values of average magnetic flux density increases with the increase of applied current at different initial gap distances. It shows that the magnetic flux density increases as the applied current increases, which agree with the result presented by Mazlan et al.^25^. When the applied current is higher than 1.0 A, the values of the simulated magnetic flux density become higher than those of the measured, which could be attributed to the magnetic flux leakage and the magnetic saturation in the measurement.

## Experiment

**Figure 7:**
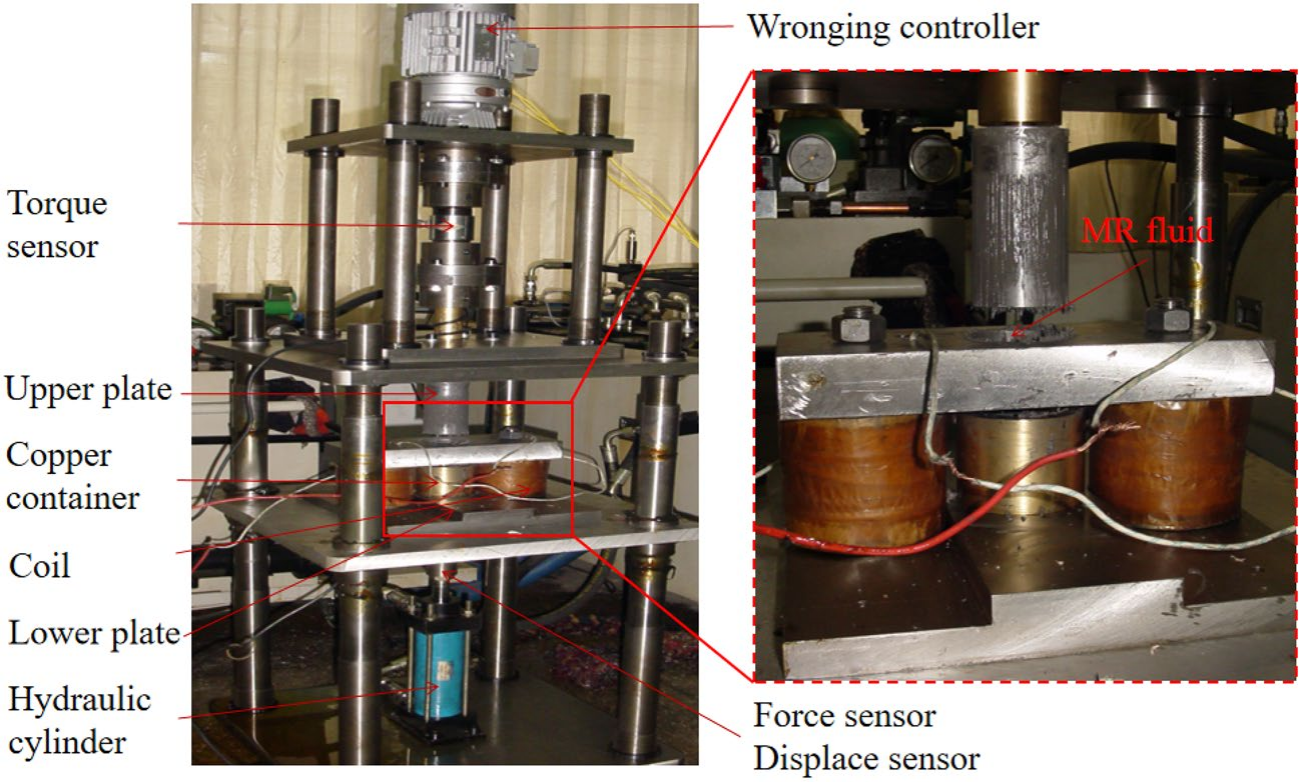
Photo of experimental setup for compression and shear after squeeze performance of the MR fluid.

According to Fig. 1, a equipment setup is fabricated, as shown in Fig. 7. In this experiment, the MR fluid (MRF-2035) purchased from Ningbo Shangong Co. Ltd, China was used, which is based on carbonyl iron particles and silicone oil. Its property of the MR fluid was disclosed in an earlier work^4^.

In the compressive experiment, the gap distance was first adjusted to the initial gap distance. The initial gap distance between the two parallel plates has been set to 1.5 and 2.5 mm, respectively. Then the lower plate was moved up at a speed of 100 μm/s to squeeze the MR fluid after different magnetic flux density values (0.1, 0.2, 0.3, 0.4, and 0.5 T) were applied, respectively. During the compression, the applied current remained constant and was turned off after the compression. The compressive stress *σ* can be represented as

11 $$\sigma = \frac{F}{A}$$

where *F* is the tested compressive force and *A* is the area of the upper plate. The compressive strain *γ* can be represented as^4^

12 $$\gamma = \frac{1}{{h_{0} }}\left( {vt - \frac{F}{k}} \right)$$

where *v* is the compressive speed, *t* is the moving time of the lower plate, and *k* is the elastic factor of the force sensor.

In the shear after squeezing experiment, the initial gap distance was set to 2.5 mm. After a magnetic field had been applied to the MR fluid, the lower plate was moved up at a speed compressive of 100 μm/s. Then the upper plate rotated and the shear rate was in the range of 0–13.8 s^−1^. The same procedure was executed in the next set of experiments at different magnetic flux density values (0.1, 0.2, 0.3, 0.4, and 0.5 T) and different compression stresses (0, 0.5, 1.5, 2.5, and 3.5 MPa), except that the initial gap distance was set to 2.5 mm. The shear yield stress of MR fluids after squeeze can be represented as^26^

13 $$\tau_{y} = \frac{3}{{2\pi r^{3} }}(T - \frac{1}{2h}\pi r^{4} \eta \cdot \Delta \omega )$$

where *r* is the radius of the upper plate, *T* is the tested torque measured by the torque sensor, *h* is the instantaneous distance between the two plates, *η* is the low field viscosity of MR fluids, and Δ*ω* is the rotational speed of the motor.

## Results and discussion

**Figure 8:**
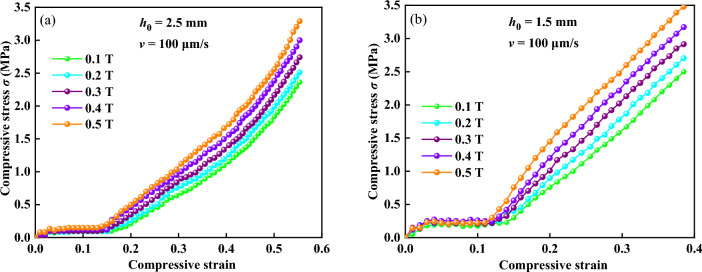
Curves of compressive stress versus compressive strain (**a**) under 2.5 mm (**b**) under 1.5 mm.

**Figure 9:**
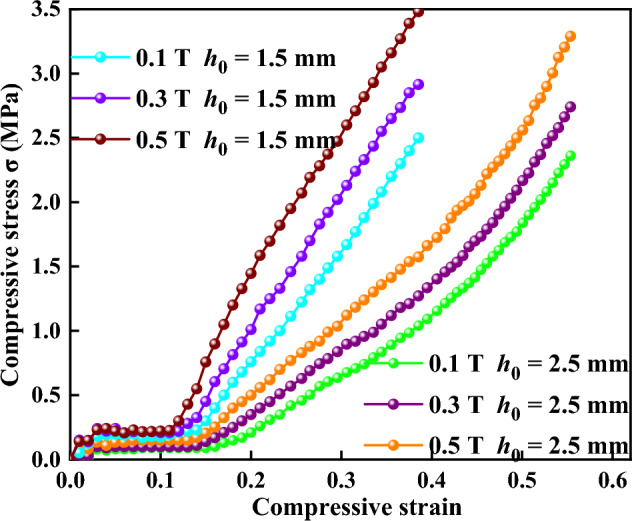
Comparison of compressive stress between 1.5 and 2.5 mm under different magnetic flux densities during compression.

The compressive stress versus the compressive strain of compressions at different initial gap distances and different magnetic flux densities is shown in Fig. 8. Five different magnetic flux densities of 0.1, 0.2, 0.3, 0.4, and 0.5 T and two initial gap distance of 2.5 and 1.5 mm were applied, respectively. Figure 8a,b show the curves of compressive stress under 2.5 and 1.5 mm, respectively. The compressive stresses under different magnetic flux densities at the beginning of compression showed almost the same values until the compressive strain reached nearly 0.148. Then, the curves of compressive stresses under different magnetic flux densities no longer well coincided and increased with the increasing compressive strain. Higher compressive stress would occur where the magnetic flux density was higher and the initial gap distance was smaller. Figure 9 shows the comparison between two different initial gap distances (2.5 and 1.5 mm) under different magnetic flux densities during compression. The curve at *h*_0_ = 1.5 mm was steeper than that at *h*_0_ = 2.5 mm. It means that a smaller initial gap distance could obtain higher compressive stress at the same strain, which is the opposite of the results of Mazlan et al.^11^ and is in agreement with the results of Guo et al.^13^. They both found that the high compressive stress occur when the compressive strain and applied current are high. However, Mazlan et al. exhibit that there are larger compressive stress values for the larger initial gap size at the same strain, and Guo et al. show that smaller gap distance generates larger compressive stress at the same strain. MR fluids are usually considered as Bingham fluids with high viscosity^27–29^. It is believed that the normal force *F* is determined by the magnetic field strength *H*, the initial gap distance *h*_0_, and the compressive strain *ε* with a relation as *F* ∝ *H*^*2*^/*h*_0_(1 − *ε*)^2^^14,30–33^. This means that a higher magnetic flux density, a smaller initial gap distance, or a larger compressive strain corresponds to a larger normal force and compressive stress.

**Figure 10:**
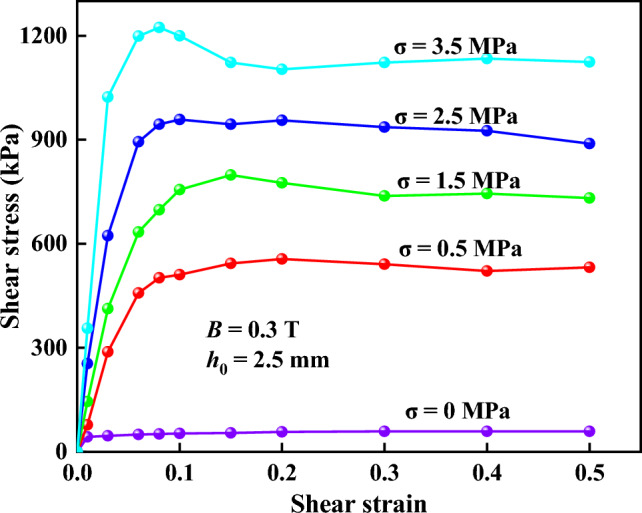
The curves of shear stress of the MR fluids after squeeze and without compression.

The shear stresses of the MR fluids after squeeze at a magnetic flux density of 0.3 T, an initial gap distance of 2.5 mm and different compressive stresses were shown in Fig. 10. It showed that the shear of the MR fluid after squeeze could be divided into two processes. In the first process, the shear stress first increased almost linearly and then increased slowly to a peak value with shear strain. This means the highest interactions between particles along the magnetic field. The peak value is considered as the shear yield stress of the MR fluid. This process can be called a yield process of MR fluids. After the yield process, the shear stress changed gradually and the shear strain increased quickly, which reflects the dynamic equilibrium process of MR fluids after shear yield stress is reached. This process can be called a post-yield process of MR fluids. Moreover, Fig. 10 showed the shear yield stress of the MR fluid without compression was only 47 kPa. However, the shear yield stress of the MR fluid after squeeze was 788 kPa at a compressive stress of 1.5 MPa, 17 times of yield stress without compression.

**Figure 11:**
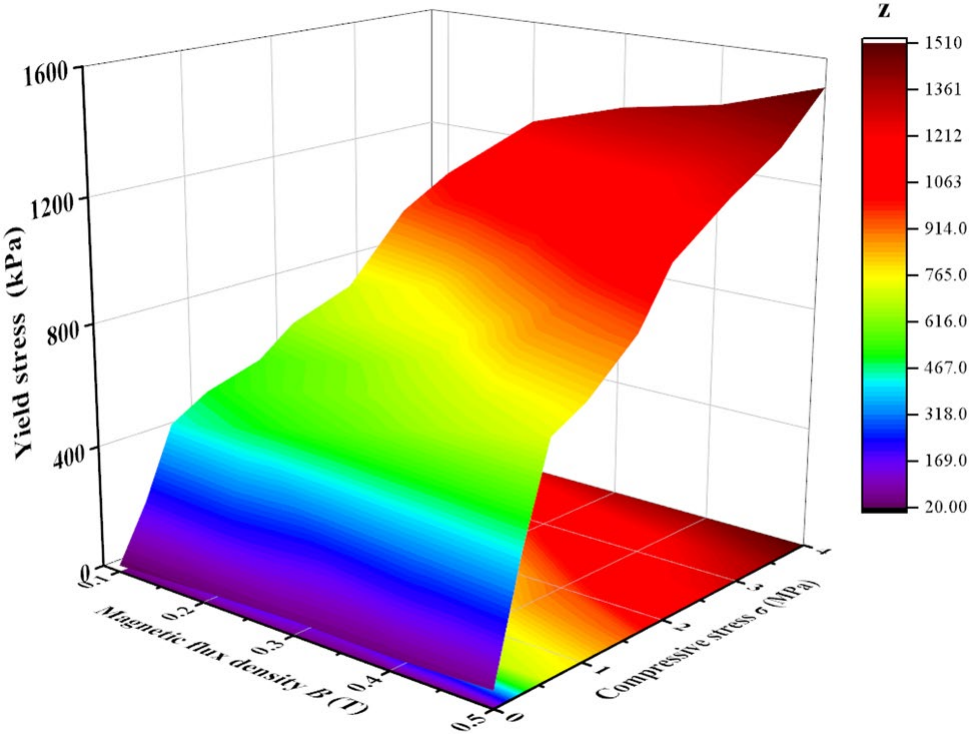
Relationship of 3D between yield stress, magnetic flux density, and compression stress.

**Figure 12:**
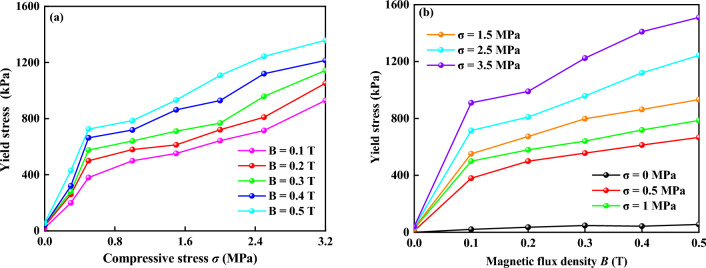
(**a**) Yield stress versus compression stress under different magnetic flux densities. (**b**) Yield stress versus magnetic flux density under different compression stresses.

The experimental results of shear yield stress after the squeeze for MR fluids at different magnetic flux densities (0–500 mT) and compressive stresses (0–3.5 MP) were shown in Fig. 11. It showed the relationship between yield stress, magnetic flux density, and compression stress. To interpret the results more clearly, the data was extracted to form the two-dimensional graph, as shown in Fig. 12. It showed the shear yield stress after the squeeze of MR fluids increased quickly with the increase of compression stress and magnetic flux density. The designed MR performance test device can obtain the shear yield stress of 1224 kPa, and 1510 kPa under the magnetic flux density of 0.3 T and 0.5 T, respectively, at the compression stress of 3.5 MPa, as shown in Fig. 12b. Similar results have been obtained in the magnetic field-dependent MR fluids, where a thin metal slice was plugged into the MR fluids to measure the shear yield stress^10^. The shear yield stress mainly depends on the compressive strain, the shearing surface dimensions, initial gap distance, magnetic flux density, and the compressive stress. This observation was comparable to those reported by Zhang et al.^10^. The shear yield stress of the proposed MR performance test device is about 2 times that by Zhang et al.^10^ under the same magnetic flux density of 0.325 T and compression stress of 3.5 MPa. The phenomenon that the shear yield stress was greatly increased after the squeeze is called the squeeze-strengthen effect^8,10^. The formation of thick strong columns/body centered cubic structures during compression enhances the shear yield stress of MR fluids in the magnetic field^9^. When the magnetic particles contact under compression, friction will happen^10,19^. Based on tribology theory, the shear yield stress *τ* can be defined as *τ* ∝ *σC*/(1 − *C*^2^]^1/2^, where *C* is the coefficient of friction and *σ* is the compressive stress^10,19^. The results show that when the distance between particles reaches a certain critical value, the contribution of friction increases greatly. Moreover, the shear yield stress was found to only increase slightly with the increase of magnetic field during compression^8^. In short, the aggregation of chain structures, especially the frictional effect in close-contact particles, results in a great improvement in compressive properties. It is promising for applications in the testing performance of MR devices based on squeeze-shear mode, for example, MR damper and MR clutch/brake.

## Conclusion

In this study, a test equipment was designed and built to perform investigations on the behaviours of compression and shear after squeeze of MR fluids. Mathematical expression of magnetic flux density in the region of MR fluid was established. A simulation analysis of the designed magnetic circuit of the test device based on two-coil mode and single-coil mode was performed and compared. The test equipment was used for compression and shear after squeeze tests on the MR fluids under different magnetic fields and different initial gap distances at a compressive speed of 100 μm/s. The results showed that under the same applied magnetic field strength, the smaller the initial gap distance, the greater the compressive strain and the greater the corresponding compressive stress. Larger compressive stress or a higher magnetic flux density corresponds to a higher shear yield stress. These results agree to the other results for MR fluids, which show that the test equipment could be used to studying the compression and shear after squeeze performances of MR fluids.

## Data Availability

All data generated or analyzed during this study are included in this manuscript.
